# VEXAS syndrome

**DOI:** 10.1007/s12185-024-03799-9

**Published:** 2024-05-31

**Authors:** Hideaki Nakajima, Hiroyoshi Kunimoto

**Affiliations:** Department of Stem Cell and Immune Regulation, Yokohama City University Graduate School of Medicine, 3-9 Fuku-Ura, Kanazawa-Ku, Yokohama, 236-0004 Japan

**Keywords:** VEXAS syndrome, UBA1, Inflammation, Myelodysplastic syndrome

## Abstract

VEXAS syndrome is a recently identified, adult-onset autoinflammatory disease caused by somatic mutations in *UBA1*. *UBA1* is an X-linked gene encoding E1 ubiquitin activating enzyme and its mutation in hematopoietic stem and progenitor cells leads to their clonal expansion and myeloid-skewed differentiation. UBA1 mutations in VEXAS are clustered at the second methionine (p.Met41), eliminating UBA1b isoform translated from p.Met41. Loss of UBA1b impairs ubiquitination and activates innate immune pathways, leading to systemic autoinflammation manifested as recurrent fever, chondritis, pulmonary involvement, vasculitis, or neutrophilic dermatitis. VEXAS syndrome is frequently associated with hematological disorders such as myelodysplastic syndrome (MDS), plasma cell dyscrasia and venous thromboembolism. Macrocytic anemia/macrocytosis and vacuoles in myeloid/erythroid precursors are prominent features of VEXAS syndrome, and their presence in patients with autoinflammatory symptoms prompts physicians to screen for *UBA1* variant. Treatment of VEXAS syndrome is challenging and no consistently effective therapies have been established. Anti-inflammation therapies including glucocorticoids and anti-interleukin-6 have shown limited efficacy, while azacytidine and JAK inhibitors such as ruxolitinib were found to induce favorable, mid-term responses. Hematopoietic stem cell transplantation is the only curative option for VEXAS and should be considered for younger, fit patients with poor prognostic factors or recalcitrant symptoms.

## Introduction

VEXAS (vacuoles, E1 enzyme, X-linked, autoinflammatory, somatic) syndrome is a recently identified, novel class of autoinflammatory disease caused by somatic mutations in *UBA1* in hematopoietic stem and progenitor cells (HSPCs) [1]. *UBA1* is an X-linked gene encoding E1 activating enzyme required for initiation of all cellular ubiquitin signaling [2–5]. The mutation of *UBA1* in HSPCs leads to clonal expansion of mutant clones in the bone marrow (BM), resulting in myeloid-skewed differentiation and abnormal activation of innate immune pathways causing systemic auto-inflammation. Patients with VEXAS syndrome not only demonstrate auto-inflammatory symptoms such as fever, polychondritis, dermatitis, lung lesion, and vasculitis, but are also associated frequently with hematologic disorders such as myelodysplastic syndrome (MDS) or plasma cell dyscrasia. Because of its pathogenesis and clinical manifestation, VEXAS syndrome can be regarded as a unique disease entity at the nexus of hematology and rheumatology. This review will overview clinical and hematologic features of VEXAS and summarize rapidly accumulating knowledge on genetics, pathophysiology, and therapeutic management of VEXAS syndrome since its discovery in 2020.

## Genetics of VEXAS syndrome

**Fig. 1:**
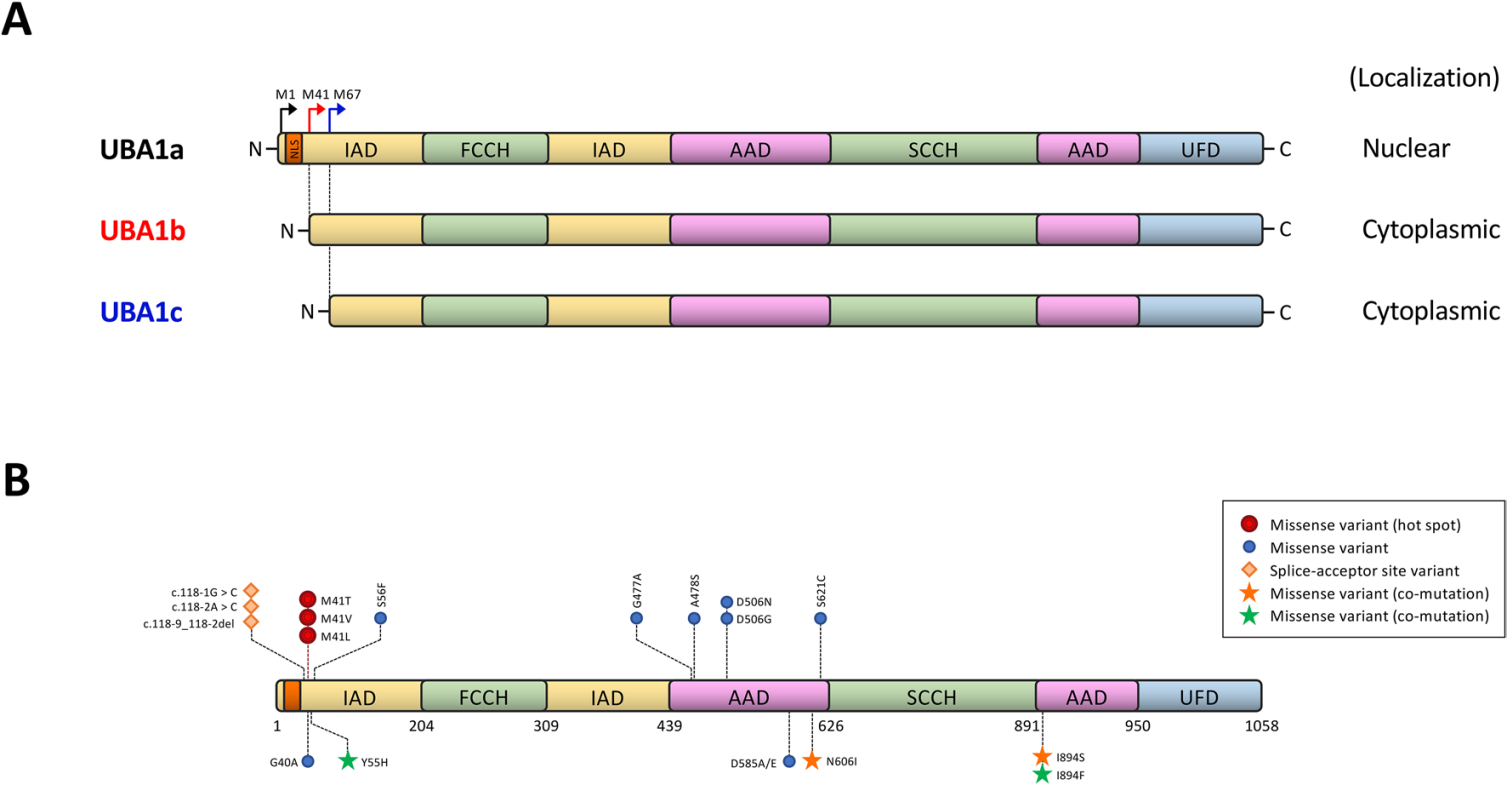
Structure and variants of UBA1 gene. **A** Structure of UBA1 and its three isoforms. UBA1 consists of five functional domains: inactive adenylation domain [IAD], first catalytic cysteine half domain [FCCH], active adenylation domain [AAD], second catalytic cysteine half domain [SCCH], and ubiquitin fold domain [UFD]. IAD and AAD adenylate the first ubiquitin, and transfer ubiquitin to the catalytic cysteine domains to form a thioester bond. UBA1a preferentially localizes in nucleus due to the nuclear localization signal (NLS) at the N-terminus, while UBA1b and UBA1c are cytoplasmic. **B** Reported variants of UBA1 in VEXAS syndrome. Orange or green stars show co-existence of two missense variants in the same patient.

In wild-type cells, UBA1 protein is expressed in two isoforms, UBA1a and UBA1b, translated from the first methionine (p.Met1) and the second methionine (p.Met41), respectively (Fig. 1A). UBA1b is exclusively located in cytoplasm due to the loss of nuclear localization signal (NLS) present in UBA1a. Initial study reported p.Met41 on exon 3 as a hot spot for *UBA1* mutation in VEXAS syndrome. The mutations substitute p.Met41 with threonine (c.122 T > C), valine (c.121A > G), or leucine (c.121A > C), leading to a loss of UBA1b and generation of catalytically impaired, novel cytoplasmic variant, UBA1c, translated from the third Methionine (p.Met67) (Fig. 1A).

Although p.Met41 variants are most prevalent (90–95%) in VEXAS syndrome, mutations at other sites have been reported in recent literatures [6–12] (Fig. 1B). Several splice-acceptor site variants have been identified in the 5’-intron/exon boundary of exon 3 (c.118-1G > C and c.118-2A > C), leading to an isoform shift from UBA1b to UBA1c similarly as p.Met41 variants [7, 11]. Non-p.Met41 missense variants have been reported at p.Tyr55 and p.Ser56 close to p.Met41, as well as p.Gly477, p.Ala478, p.Asp506, p.Asp585, p.Ser621 and p.Ile894 in active adenylation domain (AAD) [7, 9, 10, 12] (Fig. 1B). Interestingly, p.Ile894Ser and p.Ile894Phe variants were found to co-exist with p.Asn606Ile and p.Tyr55His, respectively in the same patient [10] (Fig. 1B). Non-p.Met41 variants do not affect UBA1 isoform expression but are instead considered to influence UBA1 functions through impairing the catalytic activity in temperature-dependent manner, or affecting ATP binding, UBA1 thermolability, or ubiquitin transfer from UBA1 to the E2 enzyme. Overall, the most frequent mutations associated with VEXAS syndrome are p.Met41Thr (49%), p.Met41Val (26%), and p.Met41Leu (19%), while splice site mutations account for approximately 5% of cases and the remaining variants are less than 1%.

Clonal expansion of HSPCs with *UBA1* mutation is essential in the pathogenesis of VEXAS syndrome. However, minimal clone size of *UBA1*-mutated cells required for disease initiation and/or maintenance remains obscure. Variant allele frequency (VAF) of *UBA1* mutation in peripheral blood (PB) or BM considerably varies among patients. By analyzing 40 VEXAS patients, we have reported that the overall range of VAFs in PB leukocytes was 1.7–93.3% (median 60.3%) [13]. Interestingly, VAFs of c.121A > C p.Met41Leu and c.118-1G > C were relatively high [median 73.4% (range 41.8–93.3) and 60.6% (44.3–79.2), respectively], whereas those of c.121A > G p.Met41Val and c.122 T > C p.Met41Thr spread in wide ranges [1.7–91.5% (median 42.9%) and 11.4–88.9% (50.9%), respectively] [13]. VAFs are high in HSPCs, myeloid progenitors [granulocyte monocyte progenitors (GMPs) and megakaryocyte erythroid progenitors (MEPs)], lymphoid progenitors, megakaryocytes, and peripheral neutrophils and monocytes [1]. In contrast, *UBA1* mutation is almost absent in mature T and B cells [1]. These findings indicate a strong positive selection pressure for *UBA1*-mutated clones in myeloid lineage or myeloid-skewed differentiation of *UBA1*-mutated HSPCs. Decreased peripheral lymphocyte counts in VEXAS may be a result of clonal disadvantage of *UBA1*-mutated cells in lymphoid lineage, although lymphopenia may also result from use of glucocorticoids. It is not clear at present why myeloid cells favor *UBA1*-mutant allele whereas lymphoid cells do not. Further investigation is required to elucidate precise molecular mechanism underlying this process.

## Epidemiology

VEXAS syndrome is an X-linked, adult-onset disease, and most patients are male older than 50 years of age. It should be noted that female patients harboring *UBA1* variant with monosomy X have been reported [14–16]. This suggests that loss of heterozygosity including deletion of *UBA1*, uniparental disomy, compound heterozygous mutations of *UBA1*, and skewed X inactivation may also cause female VEXAS. Patient age at diagnosis ranges from 40- to 85-years-old [1, 17–19], and only one patient under the age of 40 has been identified to possess a pathogenic *UBA1* variant [20].

By examining the presence of disease-causing *UBA1* variants among the community cohort involving 163,096 participants, the frequency of disease-causing *UBA1* variants was estimated to be 1 in 13,591 unrelated individuals (95%CI 1:7,775–1:23,758), 1 in 4,269 men older than 50 years (95%CI 1:2,319–1:7,859), and 1 in 26,238 women older than 50 years (95%CI, 1:7,196–1:147,669) [21]. All individuals with *UBA1* variant (9 male, 2 female) had VEXAS-like rheumatologic and/or hematologic manifestations, but only 55% had the classical features diagnostic to VEXAS (e.g. relapsing polychondritis, polyarteritis nodosa, Sweet syndrome). All individuals had anemia (hemoglobin: mean, 7.8 g/dL; median, 7.5 g/dL), which was mostly macrocytic (10/11 [91%]) with concomitant thrombocytopenia (10/11 [91%]). These data indicated that the presence of *UBA1* variant associated with VEXAS syndrome is a rare event in general population and does not necessarily indicate phenotypic presentation of VEXAS-associated symptoms. It is also suggested that cytopenia may precede inflammatory phenotype in individuals with *UBA1* variant. Additional studies are needed in unselected and genetically diverse populations to better define prevalence and phenotypic spectrum in general population.

## Clinical features of VEXAS syndrome

### 1) Autoinflammatory symptoms

The first paper reporting 25 VEXAS cases demonstrated that the patients shared clinical features of auto-inflammation including recurrent fever, ear and nose chondritis, cutaneous vasculitis, neutrophilic dermatosis, alveolitis, pulmonary infiltrates, and venous thromboembolism, along with progressive hematologic abnormalities, including macrocytic anemia, thrombocytopenia, and myeloid dyspoiesis, without an overt malignant hematologic condition [1]. Analysis of larger cohort revealed that the most common inflammatory symptoms in VEXAS are fever [1, 17, 18, 22] and skin lesions [18, 23], observed in 64–100% and up to 83% of the patients, respectively. Skin lesions are often diagnosed with Sweet syndrome based on the histologic findings of neutrophilic dermatosis by skin biopsy [17, 18, 22, 24]. Chondritis, particularly auricular and nasal, is the characteristic feature of VEXAS, and is present in 40–60% of the cases [1, 22]. In turn, we have demonstrated that 73% (8/11) of the male patients with relapsing polychondritis had somatic *UBA1* variants, showing that significant part of male polychondritis is associated with VEXAS syndrome [16]. The frequency of pulmonary lesions in VEXAS varies between studies, but evaluation with high-resolution computed tomography revealed abnormal lung findings in majority (70%–100%) of the patients [1, 22, 25, 26]. Venous thrombosis involving both deep and superficial vessels can occur in approximately 40% of VEXAS patients [27]. Highly elevated levels of acute phase reactants (i.e. C-reactive protein [CRP], erythrocyte sedimentation rate [ESR]) are key laboratory findings shared in all patients.

### 2) Hematologic laboratory findings

**Fig. 2:**
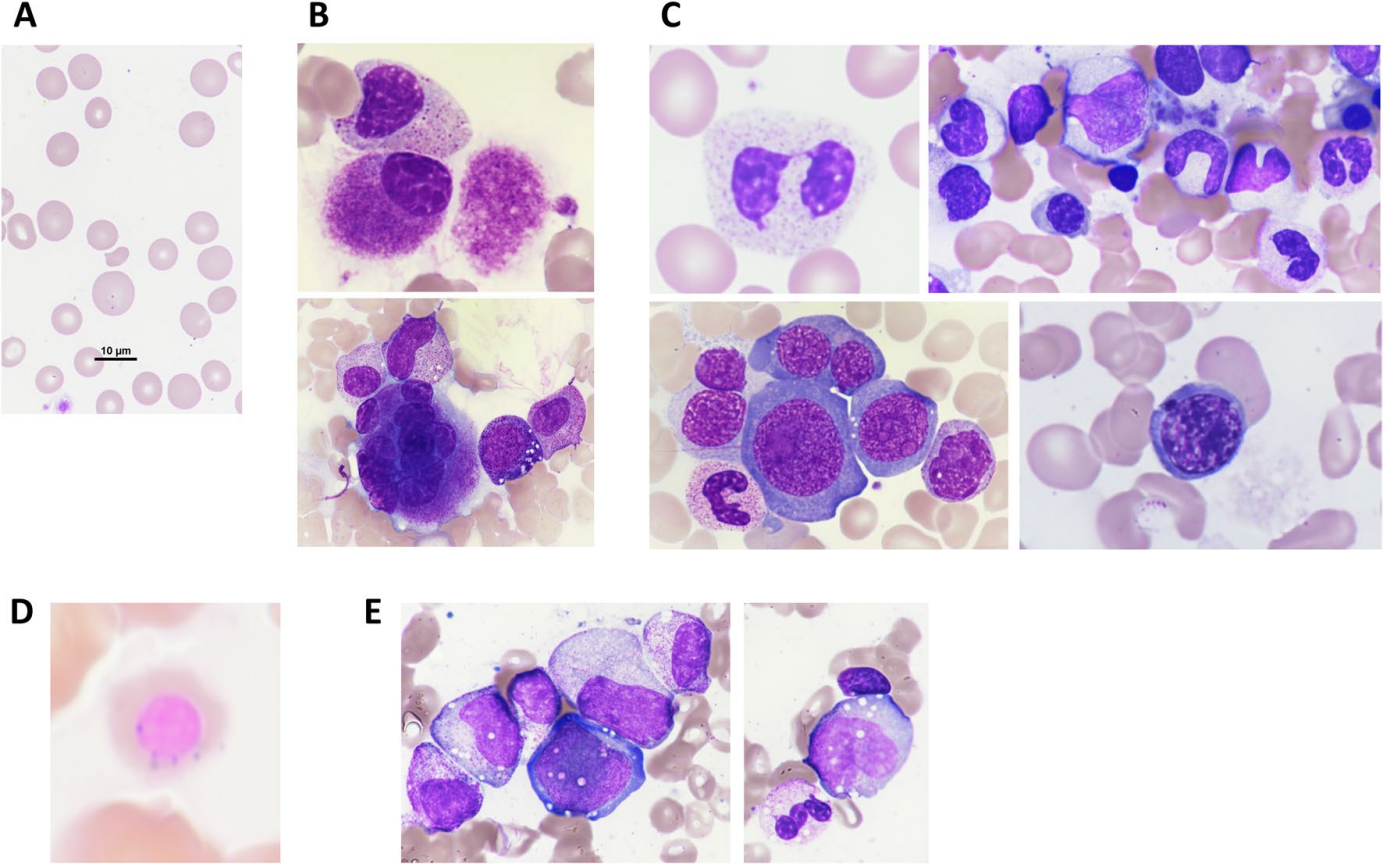
Morphology of hematopoietic cells in VEXAS patients. Representative images of PB and BM smears (adopted from Kunimoto, et al. Int J Hematol 118:494–502, 2023 [41]). **A** Macrocytic anemia in PB. **B** Megakaryocytic dysplasia. Upper panel; micromegakaryocyte. Lower panel; multinucleation of megakaryocytes. **C** Myeloid and erythroid dysplasias. Upper left; Pseudo-Pelger-Huët anomaly. Upper right; decreased granulation of myeloid cells. Lower left; megaloblastic changes and multinuclearity in erythroid cells. Lower right; karyorrhexis. **D** Ring sideroblast. **E** Cytoplasmic vacuoles in myeloid and erythroid precursors.

Hematological abnormalities are commonly observed in VEXAS patients. Typical peripheral blood findings are macrocytic anemia/ macrocytosis (90–100%) (Fig. 2A) and lymphopenia (80%) [28]. Monocytopenia is noted in 50%, while neutropenia is not common (10–20%). Thrombocytopenia is present in about 50% (45–69%) of cases, although isolated thrombocytopenia is rare. Thrombocytopenia and neutropenia become prevalent with progression of MDS.

Almost all VEXAS patients have prominent cytoplasmic vacuoles in myeloid and erythroid precursors in the bone marrow (BM) [28, 29] (Fig. 2B, C, E). Vacuoles were predominantly found in early precursors (myeloblasts, promyelocytes, and proerythroblasts). They are occasionally identified in eosinophils, monocytes, megakaryocytes, and plasma cells, but are usually absent in mature lymphocytes. BM is hypercellular in most cases and demonstrates mild to prominent myeloid hyperplasia with high myeloid/erythroid (M/E) ratio.

It should be noted that cytoplasmic vacuolization of myeloid and erythroid precursors is not a pathognomonic marker for VEXAS. Cytoplasmic vacuoles in hematopoietic precursors are associated with a number of clinical settings other than VEXAS, including copper deficiency [30], zinc toxicity [31], alcohol abuse [32], antibiotic treatment, chemotherapy, and MDS. Gurnari et al. overviewed 11,772 BM specimens and identified 24 cases with overt presence of vacuoles in hematopoietic precursors [33]. Of these, 9 cases had vacuolization in myeloid and erythroid precursors, and 2 cases out of 9 were finally diagnosed with VEXAS syndrome based on the presence of *UBA1* variant and typical clinical phenotypes. The remaining cases without *UBA1* variant showed evidence of copper deficiency, alcoholism, or MDS. These findings suggest that the unique finding of vacuolization in hematopoietic precursors in a newly diagnosed patient with myeloid neoplasms should prompt the physician to evaluate for co-occurring rheumatologic disorders and consider testing for *UBA1* variant if other causes such as copper deficiency or alcoholism have been excluded. On the other hand, lack of BM vacuolization does not necessarily rule out VEXAS syndrome, since some VEXAS cases with atypical *UBA1* variant do not manifest obvious vacuoles in the BM [11, 19].

### 3) MDS and other hematological disorders

Bidirectional link between systemic inflammation and myeloid malignancies has been described in many literatures [34, 35] and MDS is one of the most common manifestations in VEXAS. MDS has been diagnosed in approximately 50% of patients with VEXAS syndrome [1, 6, 7, 16, 19, 28, 36]. Hematopathology of BM or PB of VEXAS-associated MDS demonstrates typical features of dysmyelopoiesis (hypogranulation, pseudo-Pelger-Huët anomaly), dyserythropoiesis (megaloblastic changes, multinucleation, karyorrhexis, ring sideroblast), and dysmegakaryopoiesis (micromegakaryocytes, separated nuclei, hypolobular or mononuclear megakaryocytes) common to MDS (Fig. 2B–E). Almost all patients with an MDS diagnosis have hypercellular marrow with an increased M:E ratio (> 4:1 in more than 70% of cases). Percentage of blasts in the BM is invariably less than 5%. Approximately 20–50% of VEXAS-associated MDS patients have isolated, low to intermediate risk cytogenetic abnormalities such as del5q, del7q, del11q, del13q, del20q, -Y, or t(3;12), and no case with complex karyotype has been reported [37]. With these low-risk features, all MDS in VEXAS patients is classified to lower risk MDS by IPSS or IPSS-R. Almost all cases are diagnosed with MDS-MLD or MDS-SLD with or without ring sideroblast (RS) by WHO classification (revised 4th edition, 2017). Progression to higher risk MDS or transformation to acute myeloid leukemia (AML) is rarely seen. Genome analysis has shown that approximately in 50% of VEXAS-associated MDS, *UBA1* mutation is accompanied by isolated variants in genes related to clonal hematopoiesis (CH), most frequently DNMT3A and TET2. In contrast, somatic mutational profile does not follow that of classical MDS and none of the genes associated with poor prognosis of MDS, such as *TP53*, *RUNX1*, and *EZH2* have been found mutated in VEXAS-associated MDS. These observations further support low risk feature of VEXAS-associated MDS. Although VEXAS-associated MDS meets fundamental criteria of classical MDS (e.g. dysplasia and peripheral cytopenia), it may be regarded as a distinct disease entity of BM failure syndrome from these unique clinical and molecular features. Further studies on the molecular pathogenesis of VEXAS is essential to dissect this issue, especially on the mechanism of cytopenia whether it is due to primary BM failure caused by *UBA1* mutation or secondary to ongoing inflammation.

**Fig. 3:**
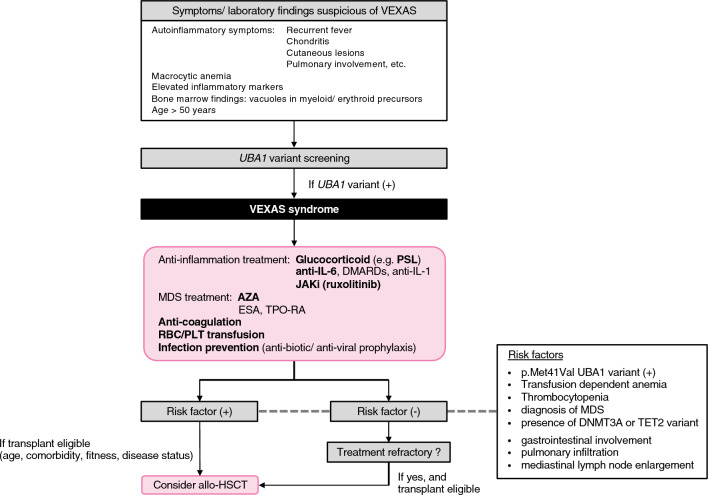
Proposed management for VEXAS syndrome based on current understandings. Screening of *UBA1* variant should be performed based on typical auto-inflammatory symptoms and laboratory findings. Scoring system for *UBA1* variant screening may be helpful for identifying VEXAS patients, although its sensitivity may not be sufficient at early stage of the disease [13]. JAKi, especially ruxolitinib and AZA are promising agents for controlling auto-inflammatory symptoms and cytopenia. Agents and managements considered to be critical in current practice are shown in bold type. Patients with risk factors (poor prognostic factors) or treatment refractory cases should be directed to allo-HSCT at an early stage. This tentative algorithm must be updated by incorporating new data from future clinical trials. *DMARD* Disease-modifying anti-rheumatic drugs; *JAKi* Janus kinase inhibitor; *AZA* azacytidine; *ESA* erythropoiesis-stimulating agents; *TPO-RA* thrombopoietin receptor agonists; *allo-HSCT* allogeneic hematopoietic stem cell transplantation

In addition to MDS, plasma cell neoplasia including multiple myeloma (MM) or monoclonal gammopathy of undetermined significance (MGUS) is observed, each in 10–20% of VEXAS patients [1, 18, 28]. Monoclonal B cell lymphocytosis is also noted to a similar frequency (10–20%). Plasma cell neoplasms may co-exist with MDS.

Thrombotic events are also common manifestation in VEXAS syndrome. Venous thromboembolism (VTE) has been reported in 40–60% of patients, while arterial thrombosis (e.g. stroke, myocardial infarction) can occur in a rare instance (1–2%) [1, 28]. Thrombotic events seem to occur early in the disease course, generally within the first 2 years after the diagnosis of VEXAS, during an active inflammatory state. Approximately 45% of patients may have recurrent clotting episodes [27].

## Clonal hematopoiesis in VEXAS syndrome

Previous studies have revealed a strong association between CH and inflammation. Systemic inflammation or inflammatory diseases have been shown to drive selection and propagation of HSPC clones with CH mutation [38, 39]. In turn, hematopoietic cells with CH mutation promote systemic inflammation by producing high levels of inflammatory cytokines such as interleukin (IL)-6 and tumor necrosis factor (TNF) α. In line with these findings, CH is observed at higher frequencies in many autoimmune or inflammatory diseases [39, 40].

Based upon these knowledge, several studies have addressed the prevalence and clinical impact of coexisting CH mutations with *UBA1* mutation in VEXAS syndrome [37, 41–43]. We and others have identified isolated CH mutations, predominantly in *DNMT3A* and *TET2*, in 50% of MDS cases with VEXAS syndrome (6 out of 12 cases in total, *DNMT3A* mutation in 2, *TET2* mutation in 2) [41, 42]. Gutierrez-Rodrigues and colleagues have screened 80 patients with VEXAS syndrome and found that 48 patients (60%) had one (35%) or more than 1 (25%) typical CH mutations concomitant with *UBA1* variant, a frequency much higher than age-matched controls [37]. CH mutations mainly involved *DNMT3A* and *TET2* (49%), and 7 patients harbored mutations in both genes. A small subset of patients had somatic mutations in *TP53* (*n* = 3), *KRAS* (*n* = 3), *NRAS* (*n* = 2), and *IDH2* (*n* = 2), with (*n* = 11) or without (*n* = 9) *DNMT3A*/*TET2* mutations. In this cohort, MDS, plasma cell dyscrasia and venous thromboembolism (VTE) were noted in 18%, 22%, and 57% of the cases, respectively. Clinically, although CH mutations did not affect phenotypic heterogeneity or disease severity of VEXAS syndrome, presence of either *DNMT3A* or *TET2* mutations increased the risk of mortality by 2 to 14-fold. Hematologic manifestations did not differ between patients with or without typical CH mutations and they were not correlated with mutational VAF. Clonal trajectory analysis revealed that *DNMT3A* mutation often precedes *UBA1* mutation in the same clone. In contrast, *TET2* or other mutations follows *UBA1* mutation, suggesting that these are likely second hits preferentially selected in an inflammatory environment created by *UBA1*-mutant cells or that they have proliferative advantage. Co-existence of independent clones with *UBA1* mutation and those with CH mutation is observed in some cases. These data indicate that the clonal landscape of VEXAS is more representative of an inflammatory disease, in which mutations in *DNMT3A* and *TET2* are enriched, as opposed to myeloid neoplasms.

## Pathophysiology

The initial study for VEXAS syndrome reported that PB cells from VEXAS patients showed decreased ubiquitylation and activated innate immune pathways [1]. Transcriptome analysis of PB monocytes and neutrophils isolated from minimally treated, clinically quiescent VEXAS patients showed highly activated inflammatory signatures in multiple pathways including TNFα, IL-6, and IFNγ, a finding that is consistent with cell-intrinsic severe myeloid inflammation. In addition, activation of pathways affecting the unfolded protein response (UPR) and integrated stress response was identified in myeloid cells.

Further analysis of BM mononuclear cells and HSPCs with single cell RNA sequence revealed that HSPCs with *UBA1* mutation are the disease driver of VEXAS syndrome [44]. The data showed that HSPCs are biased toward myeloid, especially granulocytic differentiation, and against lymphoid differentiation in VEXAS syndrome. Furthermore, activation of multiple inflammatory pathways including IFNs and TNFα occurs early in primitive HSPCs particularly in the myeloid lineage, and inflammation is prominent in *UBA1*-mutated cells. Dysregulation in protein degradation likely leads to higher stress response in VEXAS HSPCs, which positively correlates with inflammation. These data strongly suggest that *UBA1* mutations in HSPCs are the immediate driver of myeloid lineage dominance and the origin of inflammation in VEXAS syndrome.

Loss of UBA1b isoform is considered to be the primary cause for biological inflammatory phenotype in VEXAS. Ferrada et al. reported that p.Met41Thr/Val/Leu variants can produce low levels of UBA1b protein and the amount of residual UBA1b inversely correlate with the disease severity [45]. They demonstrated that p.Met41Val variant supports less UBA1b translation than either p.Met41Leu or p.Met41Thr and the presence of p.Met41Val variant is associated with inferior survival. These data indicate that the regulation of residual UBA1b translation is fundamental to the pathogenesis of VEXAS syndrome and contributes to disease prognosis.

## Prognosis and risk factors

Patients with VEXAS syndrome have an increased risk of morbidity and mortality, although the true risk of mortality compared to the general population is not clear. Current knowledge indicates that 5-year probability of survival was approximately 80% and the median survival from the onset of symptoms was 10 years [18, 22, 37].

As noted, the presence of p.Met41Val variant is associated with poor prognosis. Median survival of patients with p.Met41Val variant was 9 years and no patient with p.Met41Val lived past 12 years from symptom onset. 5-year survival of patients with p.Met41Val was 76.7%, compared to 100% with p.Met41Leu and 83% with p.Met41Thr [18, 45]. Transfusion dependent anemia, thrombocytopenia, diagnosis of MDS, and the presence of *DNMT3A* or *TET2* variant are also reported to be poor prognostic factors [37]. Another study showed that gastrointestinal involvement, lung infiltrates, and mediastinal lymph node enlargement are risk factors associated with high mortality [18]. However, *UBA1* VAF levels in peripheral blood has not been associated with increased risk of death. In contrast, ear chondritis was associated with increased survival [45].

## Therapeutic management

Treatment of VEXAS syndrome is challenging and, to date, there are no consistently effective therapies established for VEXAS. There are two main approaches for therapies; one is to inhibit inflammatory pathways or cytokines (i.e. immune suppression, cytokine inhibition, blocking DNA methylation) and the other is to target and eradicate the HSPC clones with *UBA1* mutation (hematopoietic cell transplantation). Coexistence of inflammatory and hematological (i.e. anemia, thrombocytopenia, VTE) symptoms in VEXAS often requires multidisciplinary approach for effective management, and the treatments should be tailored to each patient according to the dominant symptoms. Currently, there are no standardized guidelines for the management of VEXAS, and recommendations are based on a limited number of retrospective studies and best clinical reasoning (Fig. 3).

Glucocorticoids such as prednisolone has been the mainstay for the initial management of VEXAS. They may be effective for alleviating both inflammatory symptoms and cytopenia. However, intermediate to high dose glucocorticoids are necessary for achieving adequate symptomatic control and alternative agents are required for dosage reduction and preventing the long-term adverse events of glucocorticoids. Calcineurin inhibitors (cyclosporine, tacrolimus), anti-TNFα agents (adalimumab, infliximab), abatacept or conventional disease-modifying anti-rheumatic drugs (DMARDs) such as methotrexate, mycophenolate mofetil, azathioprine, hydroxychloroquine and colchicine are mostly effective temporarily and do not exhibit significant steroid-sparing effect [6, 17, 19, 46–48]. Owing to the progressive nature of the disease, most patients become refractory to glucocorticoids and cytopenia/inflammatory symptoms exacerbate over time.

Anti-cytokine therapies including anti-IL-1 (i.e. canakinumab, anakinra), anti-IL-6 (i.e. tocilizumab) and JAK inhibitors (e.g. ruxolitinib, baricitinib) are considered to be reasonable approaches for controlling inflammatory symptoms. Indeed, anti-IL-6 therapy using tocilizumab appears to have a favorable, although partial, suppressive effect on the inflammatory symptoms [6]. Retrospective study has shown that time-to-next treatment among patients receiving tocilizumab was 8 months [6]. In a systematic review, two-thirds of the patients could achieve glucocorticoid reduction but only 20% achieve complete response with tocilizumab [47]. We have reported that tocilizumab combined with glucocorticoids induced durable disease control at least for one year without disease progression in a small cohort [49, 50]. In contrast, outcomes of IL-1 blockade therapies in VEXAS have been equivocal. While some series have reported clinical and biochemical efficacies of canakinumab or anakinra [19, 51], others have noted ineffectiveness or intolerance to anti-IL-1 therapy [1, 52].

Signaling blockade of multiple cytokines by JAK inhibitors (JAKi) have shown significant efficacy in VEXAS. In a multicenter retrospective cohort, ruxolitinib (JAK1/2 inhibitor) was found to induce better clinical and biological responses than other JAKi such as tofacitinib, baricitinib, or upadacitinib. Complete response rate by ruxolitinib vs other JAKi was 83 vs. 18% at 3 months (*p* = 0.001) and 87% vs. 11% at 6 months (*p* = 0.002), respectively [53]. There was also a marked steroid dose reduction of 83.6% with ruxolitinib and 75% with other JAKi. Clinical efficacy of ruxolitinib was similar in patients with or without MDS. A significant increase in hemoglobin and platelet levels was observed in patients treated with ruxolitinib compared with those treated with other JAKi, and some patients achieved RBC transfusion independence. It should be noted that exacerbation of cytopenia, a common adverse event of ruxolitinib, was frequent during the first weeks of treatment. Thromboembolic complications and infections occurred in 20% and 37% of patients receiving JAK inhibitors. Although prospective studies are needed to formally establish the role of JAKi in VEXAS management, the retrospective data indicate that JAKi is a viable option in inducing biological responses and stabilization of cytopenia in VEXAS patients.

Another option to consider is azacytidine (AZA), a hypomethylating agent commonly used for higher risk MDS. AZA has been shown to be effective in controlling inflammation associated with MDS or chronic myelomonocytic leukemia [54]. A retrospective study showed that 4–6 cycles of AZA treatment improved autoinflammatory symptoms and steroid dependence in 46% (5 out of 11) of VEXAS patients over a median duration of > 1 year [55]. A phase II prospective study enrolling *UBA1*-mutated or wild type MDS patients with steroid-resistant or dependent autoinflammatory symptoms demonstrated that 75% (9/12) of patients with *UBA1* mutations achieved complete or partial responses after 6 cycles of AZA treatment, with improvement in inflammation-related manifestations and sustainable steroid-sparing effects [56]. Notably, 59% (10/17) of *UBA1*-wild-type cases also showed responses to AZA in the same study, confirming an efficacy of AZA in improving autoinflammatory features of MDS. It was suggested that patients with *DNMT3A* mutation concomitantly with *UBA1* may have increased sensitivity to AZA [42].

Allogeneic hematopoietic stem cell transplantation (allo-HSCT) may be considered as a curative option for physically fit patients generally under 65-years of age. Retrospective case studies each describing several patients have reported variable outcomes in patients receiving allo-HSCT [17, 57, 58] (Table 1). Initial study analyzing retrospectively identified VEXAS patients from HSCT registry have shown effective disease control and favorable outcome following allo-HSCT [17, 57]. However, another report have described dismal outcome after allo-HSCT, which could be due to high hematopoietic cell transplantation-specific comorbidity index (HCT-CI) score, poor disease control, continuous immunosuppressive therapy and co-morbidity of the patients before transplant [58]. A recent prospective study set the criteria for considering allo-HSCT for VEXAS patients as one of the following: (a) severe, glucocorticoid-refractory, recurrent inflammatory symptoms, (b) persistent (≥ 3 months) cytopenias, including the need for transfusions, (c) coexisting myeloid malignancy or clonal abnormalities predictive of myeloid transformation [59]. Five patients were enrolled in this study and received reduced intensity conditioning allo-HSCT. All patients were alive with disease remission after a median follow up of 9.6 months [59]. In summary, studies have indicated that allo-HSCT is a viable option in VEXAS management and should be considered at an early stage, for fit patients with either refractory cytopenias or recalcitrant inflammatory disease associated with VEXAS. Identifying poor prognostic factors of VEXAS and prospective clinical trials are necessary for selecting candidates and optimizing the timing for allo-HSCT in VEXAS management.

**Table 1:** Allogeneic HSCT for VEXAS syndrome

Median age (range)	Number of cases	Coexisting hematologic disorder	Conditioning	GVHD prophylaxis	Outcome	Reference
55.5 (46–65)	6	5/6 MDS 1/6 MF	FLU + BU, BU + CY + ATG	CsA + MTX/MMF	5/6 in CR, 1/6 died (infection)	Diarra, et al. Blood Adv. 2022, France
59 (51–67)	4	2/4 MDS	FLU + BU + thiotepa, FLU + MEL, FLU + treosulfan	CsA + MMF, CY + TAC + MMF	2/4 in CR, 2/4 died (infection)	Al-Hakim, et al. Br J Haematol. 2022, UK
61.5 (49–74)	5	1/6 MDS	FLU + MEL	PTCY	5/5 in CR	Mangaonkar, et al. Am J Hematol. 2023, Mayo

*MDS* myelodysplastic syndrome; *MF* myelofibrosis; *FLU* fludarabine; *BU* busulfan; *CY* cyclophosphamide; *ATG* anti-thymocyte globulin; *MEL* melphalan; *CsA cyclosporin A*; *MTX* methotrexate; *MMF* mycophenolate mofetil; *TAC* tacrolimus; *PTCY* post-transplantation cyclophosphamide; *CR* complete remission

Supportive therapies such as transfusion, infection prevention and anti-coagulation are critical for VEXAS management. Other supportive therapies that can be considered for improving VEXAS-associated cytopenias include erythropoietin stimulating agents (ESA) and thrombopoietin receptor agonist, which have been used in lower risk MDS and aplastic anemia, but not tested in VEXAS patients.

Overall, the effect of conventional anti-inflammatory treatments is only transient and most VEXAS patients take relapsing clinical course requiring adding or switching to new therapies. Median time to next treatment was 3.4 months for adalimumab, 3.9 months for glucocorticoids, 7.4 months for methotrexate, 8 months for tocilizumab, and 12.7 months for cyclosporine [6]. Hypomethylating agents and JAKi may be promising in controlling disease activity with median time to next treatment being 21.9 months for azacytidine and not reached for JAKi.

## Conclusion and future perspectives

VEXAS syndrome represents an emerging category of disease encompassing hematology and rheumatology. Since its discovery in 2020, a large number of case reports and analyses of patient samples have rapidly improved the clinico-biological and molecular understandings of this new disease entity. However, more functional studies using cell lines or animal models of VEXAS are needed to dissect the pathophysiological mechanism and to identify druggable targets for novel therapy. Because current therapeutic approach is based on limited retrospective data, prospective clinical trials are necessary for establishing optimal management for VEXAS, hopefully in a global setting due to its rarity and heterogeneity. It is also important to set up guidelines to make early diagnosis and take multidisciplinary, risk-adopted approach, especially in selecting candidates for allo-HSCT, which are essential for mitigating the high morbidity and mortality of VEXAS patients.

Although relationship between systemic inflammation and hematological disorders has been noted in many pathological processes, none has been integrated with causality into a unique disease entity. By taking a genotype-first approach, VEXAS was established as the first example of cross-sectional disease in ‘hemato-rheumatology’ field. It is anticipated that many more processes will be integrated into this emerging disease category through multidisciplinary approach involving experts from hematology, rheumatology, genetics, and immunology.

## Conflict of interest

HN received research funding from Nippon Shinyaku. HK has no conflict of interest.
